# Editorial Notes

**Published:** 1940

**Authors:** 


### Editorial Notes

---

**The British Red Cross Society in Bristol.**

For some years after the last war the work of the Bristol Branch of the Red Cross, under the direction of Alderman F. Sheppard, was confined to the relief of ex-service men. But three years ago it was decided, in view of the possibility of another war, that the activities of the branch should be extended. A meeting was summoned by the Lord Mayor, Alderman Moon, at which it was decided, at the request of the A.R.P. authorities, to start training courses in A.R.P. and First Aid. In the following three years over 4,000 persons took these courses of training, and received the Society's First Aid and Anti-Gas certificates. The Society also realized how essential it was that Bristol should have men and women more intensively trained in ambulance and nursing duties to meet the demands for service with the Forces or in hospitals receiving casualties—military or air raid—organized by the Ministry of Health. Three men's detachments and six women's were formed, and now number over 500 trained personnel. When war began in September, it was immediately evident that much larger premises were required if comforts for the troops at home and abroad were to be made, stored and distributed. Through the kind offices of the Chairman of the St. John Ambulance Brigade eminently suitable premises were made available on the first floor of a large building in Lewin's Mead. It was a great honour and pleasure to the Headquarters Staff of the Society when, on the 18th November, H.M. Queen Mary not only personally opened the new premises, but herself named them "Queen Mary House." Queen Mary House now accommodates several organizations, working side by side, in close and happy co-operation. Training courses and examinations in Anti-Gas, First Aid and Home Nursing and Detachment meetings and practices go on daily. Working parties of the members of the Women's Voluntary Services meet each morning and afternoon under the direction of Mrs. Herbert Thomas to make the garments, bandages and dressings required by the authorities for the hospitals. Many comforts have also been despatched to Polish refugees, and to the Finns. In another part of the building Lady Wraxall, Regional Officer for Hospital Supplies, carries on the great task of receiving from Headquarters material in bulk, which is distributed to work parties in the four counties of which Bristol is the centre, and receiving and distributing the finished articles received from the many work parties. A Hospital library, under Mrs. Charles Budgett, collects and distributes books and magazines to all the hospitals in and around Bristol where sick troops are received. Postal messages to relations in Germany are despatched from the office, and, in due course, provision will be made for a Prisoners' of War Parcel Department Though no Air Raid or Overseas casualties have yet been received in Bristol, the Red Cross has already been called upon to give help to the troops in training stationed in this area, who have been suffering severely from the exceptionally hard winter weather. At the beginning of February the military authorities asked the Red Cross to assist in equipping and staffing St. Peter's

Church Hall, Henleaze, as an extension of the hospital at Horfield Barracks. In three days' time the hospital was ready to receive forty cases, and ever since it has been dealing with a steady stream of sick men. Quite recently this branch of the Society has also been asked to help in the matter of comforts for the Medical Posts scattered all round Bristol, which have all had to be extended during the influenza epidemic. Over 50,000 articles have, in the last five months, passed out through Queen Mary House, and the Society hopes to continue to give adequate help to every call made upon it, whatever the future may bring.

**Influenza.**

WHEN a strange disorder almost simultaneously attacks nearly every inhabitant in a region, it is easy to understand how an astrological age ascribed the visitation to something flowing in from a malign comet. The epidemic that has recently visited us comformed closely to the usual main features: the attack rate was extremely high; children were only mildly affected; onset was very sudden, so that the victim who felt quite well at midday would be shivering by tea-time; and severity of symptoms and frequency of complications increased with the progress of the epidemic. In actual symptoms it was rather peculiar. The initial attack was short and often mild; following this came laryngitis and tracheitis, frequently with complete aphonia; and after five to eight days a more severe bout of fever, coryza and malaise. Many, therefore, maintained that they had had two attacks or had got up too soon. Others did not observe the initial fever and declared that the hoarseness came before the influenza.

Nothing is more remarkable about influenza than the manner in which each epidemic has its own pattern of complications. Thus with one we see bronchiolitis, with another appendicitis, with a third laryngitis, with a fourth mental depression : our recent visitation provided in the main only the common sequelae of infections of the ear and nasal sinuses, perhaps unusually numerous and severe.

**New President of the Queen Victoria Jubilee Convalescent Home.**

MR. F. C. LUKE, J.P., was unanimously elected President of the Convalescent Home at a Governors' meeting, held on 26th April, 1940, in succession to Mr. R. H. Warren, who has resigned on account of ill-health. Mr. Luke has been a valued member of the Visiting Committee for a number of years and one of the two Vice-Presidents. Mr. Warren, we are glad to hear, has consented to remain Honorary Treasurer of the Home. It is interesting to note that the Fortieth Anniversary of the Home was celebrated this year. Its financial position is most enviable and exceptional; at the close of 1939 there was an excess of income over expenditure amounting to £410. This institution illustrates that one voluntary charity, at any rate, in Bristol does not need to be in debt in order to prosper.

